# The Wiskott–Aldrich syndrome protein is required for positive selection during T-cell lineage differentiation

**DOI:** 10.3389/fimmu.2023.1188099

**Published:** 2023-06-07

**Authors:** Melissa Pille, John Avila, Guillem Sanchez Sanchez, Glenn Goetgeluk, Stijn De Munter, Hanne Jansen, Lore Billiet, Karin Weening, Haipeng Xue, Sarah Bonte, Joline Ingels, Laurenz De Cock, Eva Pascal, Lucas Deseins, Tessa Kerre, Tom Taghon, Georges Leclercq, David Vermijlen, Brian Davis, Bart Vandekerckhove

**Affiliations:** ^1^ Laboratory of Experimental Immunology, Department of Diagnostic Sciences, Ghent University, Ghent, Belgium; ^2^ Brown Foundation Institute of Molecular Medicine, Mc Govern Medical School, University of Texas Health Science Center at Houston, Houston, TX, United States; ^3^ Department of Pharmacotherapy and Pharmaceutics, Université Libre de Bruxelles (ULB), Brussels, Belgium; ^4^ Institute for Medical Immunology, Université Libre de Bruxelles (ULB), Brussels, Belgium; ^5^ ULB Center for Research in Immunology (U-CRI), Université Libre de Bruxelles (ULB), Brussels, Belgium; ^6^ WELBIO Department, WEL Research Institute, Wavre, Belgium; ^7^ Cancer Research Institute Ghent (CRIG), Ghent, Belgium; ^8^ Department of Applied Mathematics, Computer Science and Statistics, Ghent University, Ghent, Belgium; ^9^ Department of Internal Medicine and Pediatrics, Ghent University Hospital, Ghent, Belgium; ^10^ Department of Hematology, Ghent University Hospital, Ghent, Belgium

**Keywords:** T-cell development, Wiskott Aldrich syndrome, T-cell repertoire, ATO, CRISPR/Cas9, INDEL, primary immunodeficiencies

## Abstract

The Wiskott–Aldrich syndrome (WAS) is an X-linked primary immune deficiency caused by a mutation in the WAS gene. This leads to altered or absent WAS protein (WASp) expression and function resulting in thrombocytopenia, eczema, recurrent infections, and autoimmunity. In T cells, WASp is required for immune synapse formation. Patients with WAS show reduced numbers of peripheral blood T lymphocytes and an altered T-cell receptor repertoire. *In vitro*, their peripheral T cells show decreased proliferation and cytokine production upon aCD3/aCD28 stimulation. It is unclear whether these T-cell defects are acquired during peripheral activation or are, in part, generated during thymic development. Here, we assessed the role of WASp during T-cell differentiation using artificial thymic organoid cultures and in the thymus of humanized mice. Although CRISPR/Cas9 WAS knockout hematopoietic stem and progenitor cells (HSPCs) rearranged the T-cell receptor and differentiated to T-cell receptor (TCR)^+^ CD4^+^ CD8^+^ double-positive (DP) cells similar to wild-type HSPCs, a partial defect in the generation of CD8 single-positive (SP) cells was observed, suggesting that WASp is involved in their positive selection. TCR repertoire analysis of the DP and CD8^+^ SP population, however, showed a polyclonal repertoire with no bias toward autoreactivity. To our knowledge, this is the first study of the role of WASp in human T-cell differentiation and on TCR repertoire generation.

## 1 Introduction

Wiskott–Aldrich syndrome (WAS) is an X-linked primary immunodeficiency. Patients with WAS suffer from recurrent infections, eczema, thrombocytopenia, severe immunodeficiency, and an increased risk of autoimmune diseases ​ (1–3)​. WAS is caused by a mutation located in the WAS gene that encodes for the WAS protein (WASp). WASp, together with its family members (N-WASP, WAVE2, etc.) and several other actors (myosin, carmil, etc.), are classified as nucleation-promoting factors. This classification is based on the way they bind to the ARP2/3 complex, a complex needed for actin filament formation ​ (4)​. WASp is expressed in nearly all hematopoietic cells, resulting in a broad spectrum of affected hematopoietic cell lineages in patients with WAS. WASp plays a role in many different processes such as phagocytosis and chemokine-induced migration ​ (5)​. Because of its role as a regulator of the actin filament formation, WASp deficiency affects the immune synapse generation upon T-cell activation ​ (5–7). WAS patient T cells fail to proliferate and produce cytokines such as Interferon-gamma(IFNγ) and Interleukin-2(IL-2) upon aCD3/aCD28 activation. Immunodeficiency and autoimmunity are, in part, caused by defective T-cell functions ​ (5, 6, 8)​.

Because T-cell receptor (TCR) signaling is involved at several stages during T-cell development, WASp may play a role during T-cell ontogeny. Patients with WAS have a reduced number of peripheral T cells, compared with healthy individuals ​ (9, 10)​. Remarkably, this deficit is already present in patients at a very young age and is most pronounced in the naïve T-cell compartment ​ (10)​. Borte et al. (2014) reported severely reduced levels of T-cell receptor excision circles in four of the 11 newborns with WAS, which suggests a reduction in thymic output ​ (11)​. Analysis of the TCRβ repertoire revealed an oligoclonal outgrowth in the memory CD8 T-cell population, and, in one of the patients, clonotypic expansions were also found in the naïve CD8 population ​ (12, 13). This could imply that the narrowed T-cell repertoire is caused by either limited survival of peripheral T cells and/or the defective repertoire generation in the thymus. Another line of evidence for the role of WASp during T-cell development constitutes the reports of revertant mosaicism in 10% of the patients with WAS (14, 15)​. Revertant WAS T cells exhibit a polyclonal TCRβ repertoire, suggesting that the revertant mutations arose in the thymus before TCRβ rearrangements ​ (16–18)​. Finally, Laskowski et al. (2016) showed that T-cell development started from WAS-induced Pluripotent Stem cells(iPSCs) was deficient in generating CD4^+^CD8^+^ double-positive (DP) and mature CD3^+^​ T cells (19)​.

This work aims to further elucidate the role of WASp during T-cell differentiation starting from CD34^+^ hematopoietic stem and progenitor cells (HSPCs). As thymic differentiation cannot be studied directly in patients, T-cell development and, especially, the CD3 signaling–dependent β-selection and positive selection were studied using two different model systems: *in vitro* artificial T-cell organoid (ATO) cultures and humanized mice. In the ATO-culture model, we additionally studied the generation of TCRβ and TCRα repertoire. Our results point toward a role of WASp during positive selection in the thymus possibly causing the T-cell lymphopenia seen in patients with WAS. However, no evidence was found for defects in the formation of the TCR repertoire during thymic differentiation.

## 2 Materials and methods

### 2.1 Statistical analysis

All calculations and statistical analyses were performed using GraphPad Prism software version 8.4.3. The used statistical tests are indicated in the figure legends. P-values less than 0.05 were considered as statistically significant.

### 2.2 gRNA design

The IL2Rg guide RNA(gRNA) (5′-TGGTAATGATGGCTTCAACA-3′) was designed by Pavel-Dinu et al. (2019) (20). No alterations were made. The gRNAs targeting intron 1 (5′-CATGACAGTCATGGGCCCAA-3′) and exon 2 (5′-CTGGACCAAGGAGCATTGTG-3′) of the *WAS*-gene were identified using the CRISPR Guide RNA Design online tool from Benchling (https://www.benchling.com/crispr/) and verified using the Knockout Guide Design tool from Synthego (https://design.synthego.com/#/). All the guides were chemically modified and contained a 2-*O*-methyl and 3′-phosphorothioate internucleotide linkage at the three first and last bases at both 5′ and 3′ ends (Synthego, Redwood City, CA, USA).

### 2.3 Measuring indel frequencies

A minimum of 1,000 CD34^+^ hematopoietic stem cells (HSCs) or T cells were pelleted 3 days after nucleofection. The pellet was resuspended in 10 µl/10,000 cells (with a minimum of 10 µl) of the Quickextract DNA Extraction Solution (LGC, Biosearch Technologies, Berlin, Germany). The kit protocol was followed as described by the supplier. The DNA concentration was measured using a Nanodrop and altered to a concentration of 25 ng/µl. The regions of interests were subsequently PCR-amplified and Sanger-sequenced (primers and sequencing primers described in **Supplementary Table 1**.) Indel frequencies were quantified using the TIDE software (http://shinyapps.datacurators.nl/tide/) or ICE (https://ice.synthego.com/).

### 2.4 Donor template generation and AAV6 production

Donor templates WAS_exon2_-PGK-GFP (WAS_exon2_ cDNA followed by a STOP codon) and WAS2-12-PGK-GFP (WAS_exon2-12_ cDNA) were generated as described by Pille et al. (2022).

### 2.5 Human material

Male cord blood (CB) was obtained from the Hematopoietic Cell Biobank after approval by the Medical Ethical Committee of the Ghent University Hospital. Buffy coats were collected following the guidelines of the Medical Ethical Committee of the Ghent University Hospital under informed consent in accordance with the Declaration of Helsinki.

### 2.6 Flow cytometric analysis

Analysis of samples was performed on a LSR2 and Fluorescence-activated Cell Sorting(FACS) on an ARIA or Fusion-instrument (BD Biosciences, San Jose, CA, USA). Single cells were gated on the basis of FSC-Height(FSC-H) and FSC-Area(FSC-A), and live cells were gated out using Propidium Iodide(PI) or the LIVE/DEAD™ Fixable Aqua Dead Cell Stain Kit (ThermoFisher Scientific, Waltham, MA, USA). Antibodies were obtained from BioLegend (San Diego, CA, USA): CD1a PB, CD3 Pe-Cy7, CD3 APC-Cy7, CD3 BV421, CD4 PerCp-Cy5.5, CD5 PE, CD8 Amcyan, CD8 APC-Cy7, CD14 PB, CD19-APC, CD27 APC-Cy7, CD33 PE, CD34 PerCp-Cy5.5, CD34 APC, CD45 Amcyan, CD45 Pe-Cy7, CD56 BV421, CD69 Pe-Cy7, CD137 PE, interferon-γ PE, IL-2 PB, TCRαβ-PE, and TCRγδ APC; BD Biosciences (San Jose, CA, USA): CD7-FITC; ThermoFisher Scientific (Waltham, MA, USA): CellTrace™ Violet Cell Proliferation Kit; and SCBT (Dallas, TX, USA): WASp-AF647.

### 2.7 Isolation of cells

Mononuclear cells were isolated by density gradient centrifugation using Lymphoprep™ (STEMCELL Technologies, Vancouver, Canada). CD34^+^ cells were subsequently selected using the human CD34 MicroBead kit (Miltenyi Biotec, Cologne, Germany) according to the manufacturer’s instructions. The labeled cell suspension was passed three times through the magnet to obtain a purity of >95%. Sample purity was assessed on a BD LSR2 with anti-human CD34 APC and anti-human CD3 Pe-Cy7 (BioLegend, San Jose, CA, USA).

### 2.8 Cell culture conditions

CD34^+^ HSPCs were incubated in StemSpan Serum-Free Expansion Medium (SFEM) supplemented with thrombopoietin (TPO), Stemcell factor (SCF), FLT3-ligand (FLT3-L), and IL-6 (all at 100 ng/ml), StemRegenin 1 (0.75 µM), and UM171 (35 nM) (= SFEMc) (all from STEMCELL Technologies, Vancouver, Canada; except for IL-6: Tebu-Bio, Le Perray-en-Yvelines, France) at 21% O_2_ in humidified air. T cells were stimulated with 10 µl of Immunocult Human CD3/CD28/CD2 T-cell activator (STEMCELL Technologies, Vancouver, Canada) in the presence of IL-2 (10 ng/ml) in Iscove's Modified Dulbecco's Medium (IMDM) (Gibco, Invitrogen, Waltham, MA, USA) supplemented with 10% fetal calf serum (FCS; Gibco, Invitrogen), 2 mM L-glutamine (Gibco, Invitrogen), penicillin (100 IU/ml; Gibco, Invitrogen), and streptomycin (100 IU/ml; Gibco, Invitrogen) [complete IMDM (cIMDM)]. Murine-stromal cell line 5 -Delta Like Canonical Notch Ligand 4(MS5-DLL4) cells were passed through Dulbecco's Modified Eagle Medium (DMEM) (Gibco) supplemented with 10% FCS. Generated ATOs were cultured on a 0.4-μm Millicell transwell in Roswell Park Memorial Institute(RPMI) complemented with 4% B27, 30 µM L-ascorbic acid, 1% penicillin/streptomycin, 1% GlutaMAX, FLT3-L (5 ng/ml), and IL-7 (5 ng/ml) (ATO medium). Medium was replaced twice weekly.

### 2.9 Gene editing of HSPCs and T cells

Precultured CD34^+^ HSPCs were nucleofected according to the protocol of Bak et al. (2018) (21). In short, Cas9 protein (Integrated DNA Technologies(IDT), Coralville, IA, USA) was complexed with the single guide RNAs(sgRNA(s)) (molar ratio of 1:2.5) for 10 min at 25°C to form the RNP complex. Exactly 0.2 x10^6^ cells were pelleted, resuspended in the supplemented Nucleofection Solution (Lonza, Basel, Switzerland), and subsequently mixed with the RNP complex. The suspension was transferred to a cuvette and nucleofected using the Lonza 4D and the DZ100 program. After electroporation, the cells were transferred to a 48-well plate containing the Serum-Free Expansion Medium complete(SFEMc) described above and placed in the incubator or immediately transduced with 10^5^ viral genomes per cell containing the desired donor constructs. After overnight incubation, viral particles were washed away.

Ten days after stimulation of the T cells with Immunocult Human CD3/CD28/CD2 T-cell activator, the cells were harvested and nucleofected according to the following protocol. Exactly 0,4x10^6^ cells were pelleted, resuspended in the supplemented Nucleofection Solution, and mixed with the RNP complex. Using the Lonza 4D and the EH100 program, the cells were nucleofected. After nucleofection, the cells were transferred to a 48-well plate containing cIMDM with IL-2 (10 ng/ml). The next day, cells were transferred onto feeder cells for expansion until a sufficient number was present to perform functional assays.

### 2.10 Generation of artificial thymic organoid cultures

Artificial thymic organoids (ATOs) were generated as described by Seet et al. (2017) with minor alterations (22). In short, MS5-DLL4 cells were harvested using trypsin and counted. A total of 150,000 MS5-DLL4 cells per ATO were added to 7,500 CD34^+^ HSPCs in a 1.5-ml screw-cap Eppendorf. After centrifugation in a swinging-bucket centrifuge at 37°C, 300g for 6 min, the supernatant was removed, and the cells were washed with ATO medium. The supernatant was carefully removed to 5 µl of ATO, and the pellet was resuspended using a vortex. A 0.4-μm Millicell transwell insert was hydrated 3 h prior to the experiment in 1 ml of ATO medium in a six-well plate. The membrane was then removed from the six-well plate using a pincer, and the cell suspension was carefully pipetted on the membrane using a 10-µl pipet. The membrane was placed back in the well, and medium was replaced by 1 ml of fresh ATO medium. Medium was replaced every 3 to 4 days.

### 2.11 ATO harvest and analysis

ATOs were harvested at weeks 3, 6, and 9 by pipetting Magnetic-activated cell sorting (MACS) buffer to the cell drop. The organoid was held down the well using a syringe, and the medium was pass through a 50-µm nylon cell strainer. The organoid was disaggregated by pipetting 1mL of MACs-buffer vigorously on the organoid. The medium was again pass through on the cell strainer. This was repeated until the organoid had fallen apart and was lucent.

T-cell differentiation was evaluated at week 3 by staining with CD5, CD7, CD45, CD3, CD4, CD8, TCRγδ, CD56, and CD14, at weeks 6 and 9 by staining with a tube containing the markers from week 3 and a tube containing CD69, CD27, CD8, CD4, CD3, CD1, and TCRαβ. When starting from transduced HSPCs, T cells were harvested, stained for membrane markers, and were subsequently fixed and permeabilized using the Cytofix/Cytoperm Kit (BD Biosciences) before intracellular staining for WASp.

At week 3, the relevant populations were sorted out using staining with the following markers: CD5, CD7, CD34, hCD45, CD56, and CD14. At weeks 6 and 9, CD3, CD4, CD8, TCRαβ, CD56, and CD14 were used to collect the needed populations to evaluate the indel frequency.

### 2.12 *In vivo* transplantation of gene-edited HSPCs

All mice experiments were performed in accordance with the guidelines of the Ethical Committee for Experimental Animals at the Faculty of Medicine and Health Sciences of Ghent University (ref. no. ECD17/05, Ghent, Belgium). NOD scid gamma mouse (NSG) pups (maximum of 3 days old) were irradiated 8 h prior to transplantation with 100 cGy. Gene-edited HSPCs were injected intrahepatically. At 10–11 weeks after injection, the mice were sacrificed, and the bone marrow, spleen, and thymus were harvested. Single-cell suspensions were generated from these organs. Engraftment was assessed by hCD45 staining. The presence of human B cells (CD20^+^), T cells (CD3^+^), and monocytes (CD33^+^) in the spleen was analyzed using flow cytometry. Surface markers (CD4, CD8, CD3, TCRαβ, CD69, hCD45, and CD20) were used to identify the populations of interest and to sort them for indel frequency determination.

### 2.13 T-cell functionality assessment

All functional assays are based on the protocols described in Dupré et al. (2006) with minor alterations (23).

Cytokine assay: Non-Tissue Coated(NTC) flat-bottom 96-well plate wells were coated overnight with OKT3 monoclonal antibody(mAb) American Type Cell Culture (ATCC) at the indicated concentrations. The next day, excess OKT3 was removed. A total of 50,000 cells were added in cIMDM with aCD28 (10 ng/ml; BD Biosciences, Franklin Lakes, NJ, USA). Two hours later, brefeldin A Golgiplug, (BD biosciences) was added in a 1/750 final dilution. After an additional 4 hours, T cells were harvested, stained for membrane markers, and subsequently fixed and permeabilized using the Cytofix/Cytoperm Kit (BD biosciences) before intracellular staining for IFNγ, IL-2, and WASp.

Stimulation assay: The same protocol was used as for the cytokine assay except for the addition of brefeldin A. After 6 h of stimulation, cells were stained for CD3, CD4, CD8, CD69, and CD137 and for WASp after fixation and permeabilization.

Proliferation assay: enchanced green fluorescence protein (eGFP^+^) and eGFP^−^ cells were labeled with the CellTrace™ Violet Cell Proliferation Kit according to the manufacturer’s protocol. Brief, 1*10e^6^ cells were labeled with 1 µl of a CellTrace™ working solution (lyophilized CellTrace resuspended in 200 µl of dimethyl sulfoxide(DMSO)) in 1 ml of Phosphate-buffered sodium(PBS). A total of 50,000 labeled cells were then added to OKT3-coated 96-well plate together with aCD28 (10 ng/ml) for 6 days. At day 3, new cIMDM was added. Cells were harvested and stained with CD3, CD4, and CD8 and for WASp after fixation and permeabilization using the Cytofix/Cytoperm Kit.

### 2.14 TCR repertoire analysis

For the TCR analysis, the populations of interest were sorted from *in vitro* ATO cultures: 45 ATO’s transduced with *WAS*_exon2_-PGK-GFP or *WAS*_2-12_-PGK-GFP were harvested at week 9 and sorted for eGFP^+^ CD3^−^ DP, eGFP^+^ CD3^+^TCRαβ^+^ DP, and eGFP^+^ CD3^+^TCRαβ^+^CD8^+^ populations.

RNA extraction was carried out using the RNeasy Micro Kit (Qiagen, 217084), followed by a template-switch anchored Realtime(RT)-PCR. High-throughput sequencing was performed as previously described ​ (24)​. CDR3 sequence were extracted from raw sequencing fastq files after aligning the reads to reference V, D, and J genes from TRA or TRB loci of GenBank database using the MiXCR software (version 3.0.12) ​ (25)​. CDR3 sequences were analyzed using VDJtools software version 1.2.1 ​ (26)​. Out-of-frame sequences were excluded from the analysis, as well as non-functional TRA and TRB segments using IMGT (the international ImMunoGeneTics information system^®^) annotation. CDR3 sequences containing TRDV sequences were filtered as well, except for the analysis where the amount of TRDV1 containing sequences was quantified. Cumulative gene segment plots were generated using the output from CalcSegmentUsage function from VDJtools software. Tree maps were generated using the Treemap package (version 2.4-3) in RStudio, grouping TRAV and TRAJ segments according to their locus position. D75 repertoire diversity metric(s) were calculated by measuring the percentage of clonotypes required to occupy 75% of the total TCR repertoire. Other diversity readouts were obtained from the re-sampled file generated using CalcDiversityStats function. CDR3α and CDR3β apex region and cysteine usage were determined following previously described indications ​ (27)​. Percentages of hydrophobic doublets in CDR3α and CDR3β sequences were obtained by calculating the percentage of sequences using any of the 175 amino acid doublets previously identified as promoting self-reactivity ​ (28)​. Physicochemical properties were computed on the five central amino acids from the CDR3 sequences using CalcCdrAaStats function from VDJtools software.

## 3 Results

### 3.1 Exon 2–CRISPRed T cells behave as WAS patient T cells

**Figure 1:**
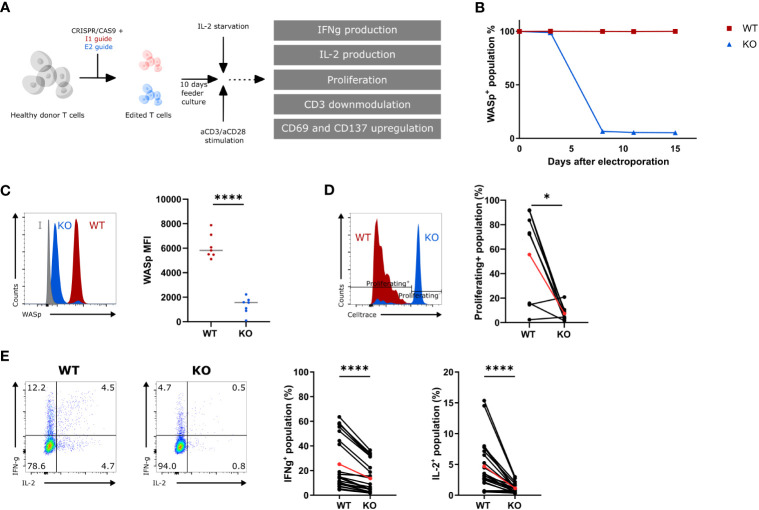
Functional assessment of WAS E2 gRNA CRISPRed T cells (KO) **(A)** Experimental outline to create and assess the functionality of WAS KO T cells. **(B)** WASp expression in intron 1 (I1 guide, WT) and exon 2 gRNA (E2 guide, KO) CRISPRed T cells over time (n=1). **(C)** Left: Representative histogram plot showing WASp expression in WT and KO T cells 10 days after nucleofection. Isotype control is shown in grey. Right: WASp expression (shown mean fluorescence of intensity (MFI)) from WT and KO T cells 10 days after nucleofection (n=7 from 7 different donors; **** p<0.0001; two-tailed paired t test). **(D)** Left: Representative histogram showing proliferation of WT and KO T cells assessed by CellTrace Violet dye dilution after 6 days of stimulation with 1.25µg/mL aCD3 and 10µg/mL aCD28. Right: Proliferating+ population in stimulated WT and KO T cells 6 days after stimulation (n=8, 4 donors; *p<0.05; two-tailed paired t test). **(E)** Left: Representative plot of IFNγ- and IL-2 production in WT and KO T cells (gated on hCD45+) after 6h stimulation with 2.5µg/mL aCD3 and 10µg/mL aCD28. Right: IFNγ+- and IL-2+-population in stimulated WT and KO T cells after 6h (n=24; 7 donors; ****p<0.0001; two-tailed paired t test). Red points in **(D, E)** represent the mean.

A gRNA targeting exon 2 (E2) of the WAS gene was designed to generate WAS knockout (KO) hematopoietic cells. This gRNA was tested side by side with a gRNA targeting intron 1 (I1), as a wild-type (WT) control. Healthy donor T cells were nucleofected with an E2-RNP or an I1-RNP and kept in culture for 10 days (**Figure 1A**). The level of WASp was measured at several time points to determine the kinetics of WASp degradation. WASp expression was no longer detectable 7 days after E2-RNP treatment (KO population) in >80% of the T cells, whereas I1-RNP–treated cells had unaltered levels of WASp (WT population) (**Figure 1B**). KO T cells show a significant drop in WASp Mean Fluorescence Intensity(MFI) at day 10 compared with WT T cells (1322 ± 262.7 vs. 6137 ± 376.4) (**Figures 1B, C**). The histograms in **Figure 1C** show two peaks for the intracellular WASp staining of which the small peak is of the same brightness as the WT (representing the unedited cells) and the larger peak which is nonoverlapping with the WT peak and is clearly less bright. This peak is assumed to represent the edited KO cells, although this staining is somewhat brighter than the isotype control. Targeted cells contain in >80% of the edits frameshift indel mutations in de second exon of the WAS-gene (data not shown) and are therefore unable to produce a WASP protein that binds our WASP-specific antibody. To investigate the functional defect in KO T cells after gene editing, the ability to proliferate, to produce IL-2 and IFNγ, and to downmodulate CD3 and upregulate activation markers was measured in response to suboptimal aCD3/aCD28 stimulation (**Figures 1A, D–E**; **S1A, B**) (23). KO T cells showed a decreased ability to proliferate compared with WT T cells upon stimulation (7.6% ± 2.2 compared with 55.7% ± 13.4 proliferating cells) (**Figure 1D**). Next, KO T cells show a two- and three-fold reduction in IFNγ and IL-2 production, respectively, compared with WT T cells (IFNγ: 13.6% ± 2.6 vs. 25.1% ± 4.4; IL-2: 1.1% ± 0.15 vs. 4.7% ± 0.8) (**Figure 1E**). Last, the KO T cells showed a somewhat weakened CD3 downmodulation upon activation (77.1% ± 2.7 vs. 58.65 ± 2.8 CD3^+^ cells in KO and WT T cells, respectively) and showed a diminished capacity to upregulate activation markers such as CD69 and CD137 (CD8^+^CD137^+^: 61.3% ± 5.1 vs. 78.2% ± 2.4; CD8^+^CD69^+^: 63.6% ± 6.4 vs. 75.8% ± 4.1; CD4^+^CD69^+^: 54.9% ± 5.5 vs. 68.6% ± 4.0) at suboptimal stimulatory conditions (**Figures S1A, B**). Together, these data demonstrate that E2-RNP–CRISPRed cells lack WASp and show functional defects similar to those described for WAS patient T cells (23). Therefore, KO cells generated using E2-RNPs provide a suitable *in vitro* surrogate for primary WAS patient cells, which are challenging to obtain and ethically more difficult to use.

### 3.2 WASp KO HSPCs are defective in the positive selection and differentiation toward single-positive T cells in ATO cultures

**Figure 2:**
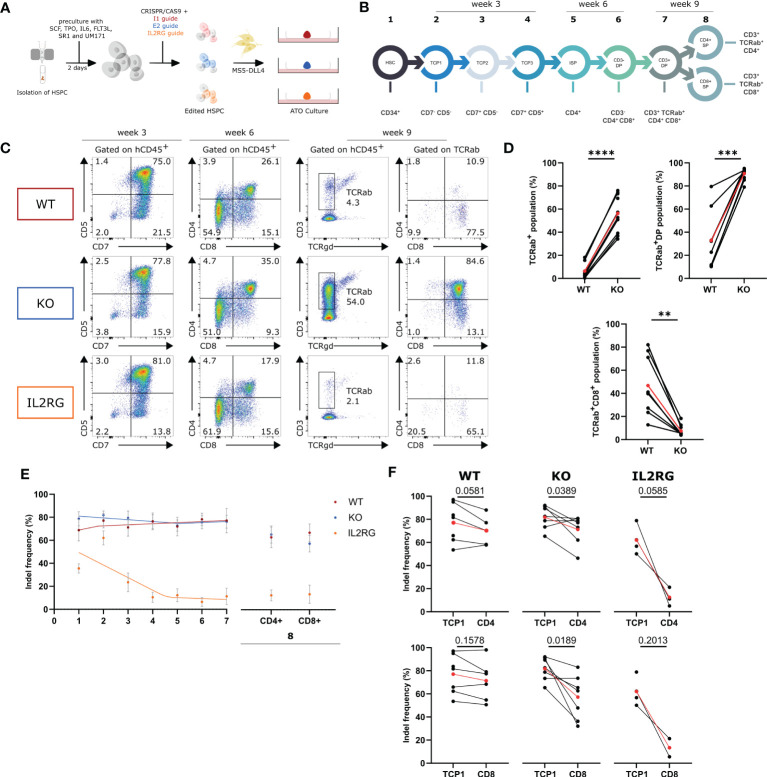
*In vitro* T-cell differentiation with Intron 1 (WT), Exon 2 (KO) and IL2RG HSPC in ATO cultures **(A)** Experimental design of generation of ATO cultures with WT, KO and IL2RG CRISPRed T cells. **(B)** Schematic representation of populations of interest in the T-cell differentiation sorted at week 3, 6 and 9 of culture. **(C)** Representative plots of the populations of interest during T-cell differentiation of WT, KO and IL2RG HSPCs at 3, 6 and 9 weeks of ATO culture. **(D)** TCRαβ+, TCRαβ+CD3+DP and TCRαβ+CD3+CD8+ populations in WT (left) and KO T cells (right) at 9 weeks of culture (gated as described in C). (n=8 of 3 experiments with 2-4 technical replicates per experiment; two-tailed paired t test). **(E)** Indel frequencies determined by Tide analysis of the populations of interest in the CD4 and CD8 T-cell development. Data is presented as mean±SEM with best fitted curve (n=7 for WT and KO; n=4 for IL2RG). **(F)** Indel frequencies at T-cell progenitor 1 (TCP1) stage (left) and CD4+ SP or CD8+ SP (right) stage for WT, KO and IL2RG. (n=7 for WT and KO; n=4 for IL2RG; exact p values in Figure; two-tailed paired t test). Red points in **(D, F)** represent the mean.

It was shown above that the E2-RNPs successfully generated WASp KO T cells. Because of the lack of WAS patient stem cells to investigate the role of WASp in the T-cell differentiation, KO HSPCs were generated using the above-described gRNA. HSPCs were isolated from male (WAS is an X-linked PID) CB and cultured in cytokine-rich medium for 2 days. Next, the cells were nucleofected with the I1-RNP or the E2-RNP to generate WT and KO HSPCs, respectively. IL2RG KO HSPCs, known to be unable to differentiate into the T-cell lineage, were generated using CRISPR technology as an additional control ​ (25)​. The edited HSPCs were subsequently mixed with MS5-DLL4 cells and cultured together as ATOs for 9 weeks (**Figure 2A**). At several time points, populations of interest in the T-cell differentiation were analyzed with flow cytometry, and, after sorting the populations, genomic DNA was isolated and analyzed for insertions and deletions. The input HSPCs are the starting point of the differentiation sequence described in **Figure 2B** (population 1). At week 3, the T-cell progenitor 1 [TCP 1 (CD7^−^CD5^−^)], TCP 2 (CD7^+^CD5^−^), and TCP 3 (CD7^+^CD5^+^) were analyzed and sorted from 3 ATOs in the WT, KO, and IL2RG conditions (populations 2 to 4 in **Figure 2B**). The ISP (CD4^+^) and CD3-DP stage (CD3^−^CD4^+^CD8^+^) were analyzed and sorted at week 6 (populations 5 and 6 in **Figure 2B**). After 9 weeks, the final two stages of the T-cell differentiation, the CD3^+^DP stage (CD3^+^TCRαβ^+^CD4+CD8^+^) and CD4^+^ single-positive (SP) (CD3^+^TCRαβ^+^CD4^+^) and CD8^+^ SP (CD3^+^TCRαβ^+^CD8^+^) were analyzed and sorted (populations 7 and 8 in **Figure 2B**). No major differences were observed at week 3 between WT, KO, and IL2RG ATOs (**Figures 2C, D**). Analysis at week 6 shows a slightly larger DP population in the KO ATOs compared with WT and IL2RG ATOs. At week 9, the KO ATOs largely contained CD3^+^ DPs in contrast to the WT ATOs (90.8% ± 1.7 vs. 32.7% ± 9.1, respectively). In addition, a 10-fold larger TCRαβ population was observed in the KO sample (56.4% ± 5.1 KO vs. 6.5% ± 2.4 WT). Both the CD3^+^ DP and TCRαβ^+^ increase led to a drop in the CD8^+^ SP stage, where the KO ATOs only generated 7.6% ± 1.5 CD8^+^ SP cells compared with 46.8% ± 9.4 in WT. This is pointed out to be a consequence of the kinetics of these cultures, which generate a single wave of differentiation that, in WT conditions, lasts for about 9 weeks. At week 9, WT DP cells have largely differentiated to CD8^+^ SP cells, which subsequently die. A partial block in positive selection induced by the WASp KO ensures that the DP cells are still present at week 9 and only slowly differentiate to CD8^+^ SP cells. Unexpectedly, we observed generation of DP T-cell precursors in the IL2RG KO condition. We hypothesized that this is caused by the lower efficiency of the IL2RG-targeted RNP, because the IL2RG gene contained indels in only 50% of the HSPCs (**Figure 2E**). Therefore, to gather additional evidence for the influence of WASp and IL2RG on the T-cell differentiation (stages 2 to 3), the indel frequencies in the populations of interest were determined (**Figure 2E**). A drop in the indel frequency in the IL2RG control was observed early during differentiation, as described before ​ (29, 30)​. This indicates that the use of indel frequencies to identify the stage at which a certain protein plays a role in the T-cell development is justified. A similar analysis on the WASp KO cells showed a moderate drop from stages 7 to 8 (i.e., DP stage to CD8^+^ SP) yet not as profound as that with IL2RG KO. However, the drop observed is significant for the CD8^+^ SP and CD4^+^ SP population (**Figure 2F**). In conclusion, we showed that indel frequencies drop in WASp KO cell at the later stages of differentiation, indicating that WAS KO cells are less efficient in passing through positive selection than WT precursor cells. However, using indel frequencies, we were unable to precisely localize the differentiation block at the DP to SP transition mediated by positive selection.

### 3.3 WASp KO HSPCs generate reduced number of CD4^+^ SP and CD8^+^ SP T cells in humanized mice

**Figure 3:**
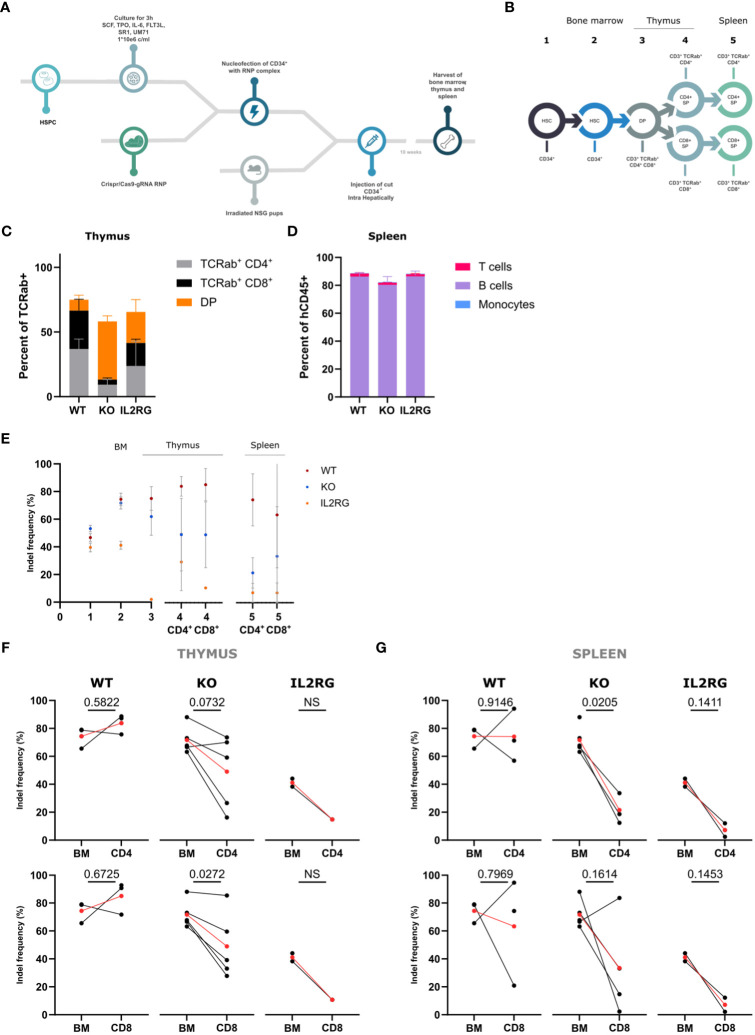
*In vivo* T-cell differentiation with Intron 1 (WT), Exon 2 (KO) and IL2RG HSPC in NSG mice **(A)** Schematic overview of the in vivo experimental set-up. **(B)** Schematic representation of populations sorted from bone marrow, thymus and spleen 10 weeks after injection. **(C)** Lineage distribution of TCRαβ+ cells in the thymus of NSG mice 10 weeks after injection (n=3 from 2 experiments; CD4+ WT vs KO: p=0.0667, CD8+ WT vs KO: p=0.0912 and DP WT vs KO: p=0.0130; two-way ANOVA with Tukey’s multiple comparison test). **(D)** Lineage distribution of human cells (hCD45+ cells) in the spleen of NSG mice 10 weeks after injection (n=4 from 2 experiments; non-significant; two-way ANOVA with Tukey’s multiple comparison test). **(E)** Indel frequencies determined by Tide analysis of the populations of interest in the CD4 and CD8 T-cell development. (n=4 for WT, n=5 for KO and n=2 for IL2RG) **(F, G)**. Indel frequencies in the Bone Marrow (BM) (left) and CD4+ SP or CD8+ SP (right) stage for WT, KO and IL2RG in the thymus **(F)** and spleen **(G)** (n=4 for WT, n=5 for KO and n=2 for IL2RG; exact p values in Figure; NS = not able to be determined; two-tailed paired t test). Data in Figure 2 are presented by mean ± SEM. Red points in **(G, H)** represent the mean. Single points have no matched value.

To evaluate the role of WASp during *in vivo* differentiation, WT, WASp KO, and IL2RG-CRISPRed HSPCs were transplanted into 3-day-old sublethally irradiated immunodeficient non-obese diabetic–severe combined immunodeficiency (SCID) Il2RG^−/−^ (NSG) pups. Healthy donor HSPCs were nucleofected with either I1-RNP (WT), E2-RNP (KO), or IL2RG-RNP. Ten weeks after intrahepatic injection of the edited HSPCs, the thymus, spleen, and bone marrow were harvested (**Figure 3A**). Both flow cytometric analysis and indel frequency measurements were performed on the populations of interest of the T-cell development described in **Figure 3B**. The starting point was again the injected edited HSPCs (population 1). In bone marrow, the CD34^+^ cells were analyzed (population 2), and, in thymus, the CD3^+^TCRαβ^+^CD4^+^CD8^+^ DP stage (population 3) was evaluated. The CD4^+^SP and the CD8^+^ SP were analyzed both in thymus (population 4) and spleen (population 5). Flow cytometric analysis of the thymi showed a significant increase in the percentage of DP cells present in the KO mice and a slightly decreased percentage of CD4^+^ SP and CD8^+^ SP T cells (**Figure 3C**). Despite the lower levels of SP T cells in the KO thymus, the percentage of SP T cells in the periphery was comparable with those in the WT and IL2RG control (**Figure 3D**). Populations 1 to 5 were sorted and analyzed for indels. As expected, a clear drop was observed in the IL2RG mice early in the differentiation process (HSC-DP stage) (**Figure 3E**). A decrease in WASp KO cells was observed and was most pronounced between populations 3 and 4 (DP-SP stage), whereas the indel frequencies in the WT mice did not diminish or even increased. A significant drop in indel frequency in the CD8^+^ SP KO T cells was already observed in the thymus (**Figure 3F**). The indel frequency in CD4^+^ SP KO cells is also decreased, but this was significant only in the periphery (population 5) (**Figure 3G**). Again, these data suggest that WASp is required for efficient differentiation from CD3^+^TCRαβ^+^CD4^+^CD8^+^ to SP cells and, to greater extent, to CD8^+^ SP cells in the thymus. The defect largely originates in the thymus, whereas the contribution of peripheral phenomena such as homeostatic expansion is minimal (**Supplementary Figure S2**).

### 3.4 WAS_exon2_-PGK-GFP (KO) HSPCs confirm the influence of WASp on positive selection

**Figure 4:**
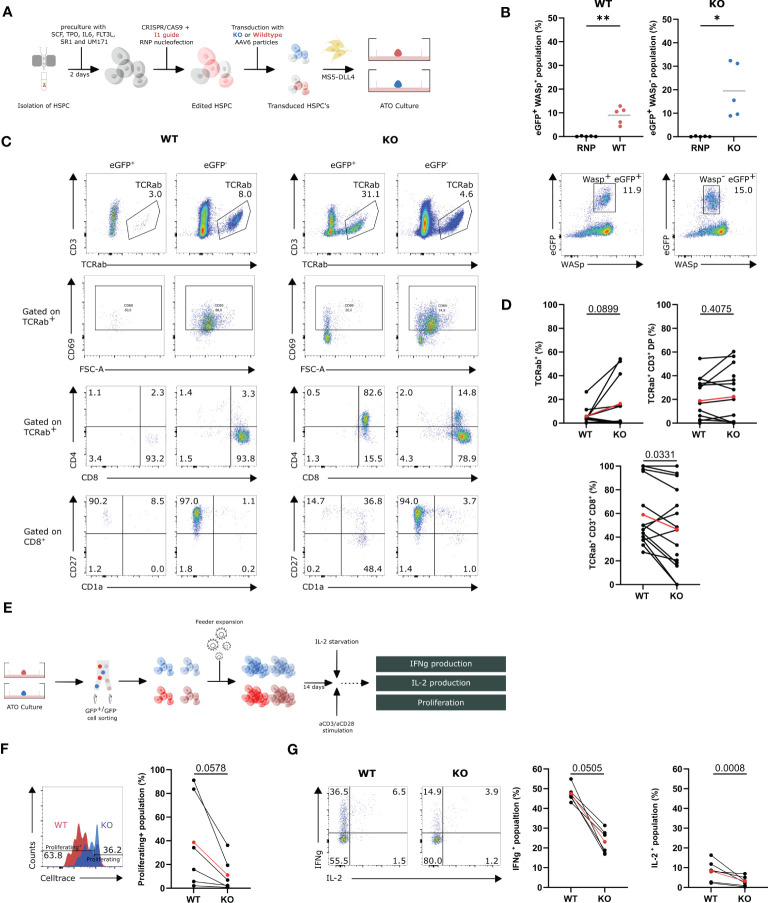
*In vitro* T-cell differentiation of WASexon2-PGK-GFP (KO) and WASexon2-12-PGK-GFP (WT) transduced HSPCs and assessment of functionality of generated T cells **(A)** Experimental design of generation of ATO cultures with WT and KO HSPCs. **(B)** Quantification (left) of eGFP+ WASp+- and eGFP+ WASp-- gene edited populations (right). (n=5, *p<0.05 and ** p<0.01, two-tailed paired t test). **(C)** Representative plots of the populations of interest in the T-cell differentiation of WT and KO HSPCs at 9 weeks of ATO culture. **(D)** TCRαβ+, TCRαβ+CD3+DP and TCRαβ+CD3+CD8+ populations in WT (left) and KO cells (right) at 9 weeks of culture gated on GFP+Wasp+ cells (WT) and GFP+WASP- cells (KO). (n=12 from 5 experiments with 2-4 technical replicates per experiment). Determined outliers were removed using the Graphpad feature “identify outliers; exact p-values in Figure; two-tailed paired t test). **(E)** Schematic representation of the generation and assessment of the functionality of WT and KO T cells. **(F)** Left: Representative histogram plot showing proliferation of WT and KO T cells assessed by CellTrace Violet dye dilution after 6 days of stimulation with 1.25µg/mL aCD3 and 10µg/mL aCD28. Right: Celltrace- population in stimulated WT and KO T cells 6 days after stimulation (n=6 from 3 experiments; exact p-values in Figure; two-tailed paired t test). **(G)** Left: Representative plot of IFNγ- and IL-2 production in WT and KO T cells (gated on hCD45+) after 6h stimulation with 2.5µg/mL aCD3 and 10µg/mL aCD28. Right: IFNγ+- and IL-2+-population in stimulated WT and KO T cells after 6h (n=6 from 3 experiments with 2 technical replicates; exact p-values in Figure; two-tailed paired t test). Data in **Figure 4** is presented as mean ± SEM. Red points in **(D, F, G**) represent the mean.

To label the edited cells for subsequent tracing and isolation, targeted integration was performed using the I1 gRNA and either a *WAS*_exon2_-PGK-GFP donor construct to create GFP^+^ WAS KO cells (KO) or a *WAS*_exon2-12_-PGK-GFP donor construct to generate control GFP^+^ WT cells (WT). HSPCs were isolated and gene-edited in the presence of these donor constructs resulting in GFP-marked KO-cells and GFP-marked WT-cells. Edited HSPCs were subsequently mixed with MS5-DLL4 and cultured as ATOs (**Figure 4A**). We observed a GFP^+^WASp^+^ (WT) population of up to 12.9% (8.9% ± 1.7) in the WT condition and a GFP^+^WASp^−^ (KO) population of 19.5% ± 5.1 in the KO condition (**Figure 4B**). Flow cytometric analysis of the populations of interest in the ATOs was performed at week 9 (**Figure 4C**). At week 9, a slight increase in TCRαβ^+^ was observed in the KO population (16.4% ± 6.6 vs. 5.8% ± 2.3) yet not significant compared with the WT (**Figure 4D**). Interestingly, no difference was observed in the percentage of CD3^+^TCRαβ^+^DP cells, but, again, there was a decrease observed in the CD8^+^ SP population when comparing the KO with WT in 15 ATOs (46.7% ± 8.8 vs. 59.0% ± 7.3) (**Figure 4D**). Remarkably, the GFP^+^ KO TCRαβ^+^ cells are phenotypically immature CD1^+^, CD27^−^, and CD69^−^, pointing toward a defective positive selection (**Figure 4C**). These data again suggests that WASp plays a role in the positive selection in T-cell differentiation.

To evaluate whether the generated T cells in ATO cultures have all the characteristics of WAS patient T cells, the ATO culture–generated T cells were subjected to functional testing. Both KO and WT T cells were harvested at week 9, sorted for GFP, and feeder-expanded for 14 days. The cells were stimulated with aCD3/CD28 (**Figure 4E**). The generated KO T cells showed a decreased ability to proliferate upon stimulation for 6 days (38.7% ± 16.0 vs. 11.2% ± 5.8) (**Figure 4F**). Next, the ability to produce IFNγ and IL-2 was tested in stimulated WT and KO T cells. KO T cells show a two-fold decrease in IFNγ production and a two-fold decrease in IL-2 production (IFNγ: 23.1% ± 2.5 vs. 47.5% ± 1.6; IL-2: 3.1% ± 1.0 vs. 8.2% ± 2.3; red points) (**Figure 4G**). Overall, we can conclude that the GFP^+^ WAS KO cells have a relatively mild defect in T-cell differentiation at the DP to SP stage, and the mature T cells display the same functional defects as WAS patient T cells.

### 3.5 The TCRα and TCRβ repertoire generated during T-cell development is polyclonal in the absence of WASp

Effective T-cell immunity is guaranteed by a diverse TCR repertoire. Several studies have shown, thus far, that the TCRβ repertoire diversity of peripheral blood T cells in patients with WAS is severely reduced. Whether the repertoire is shaped intrathymically or induced by antigen encounter in the periphery is unknown. Here, we studied the TCRα (TRA) and the TCRβ (TRB) repertoire of developing T cells generated in ATOs and thus before any antigen stimulation that may cause clonal expansions of responsive T cells. Because the TCRβ repertoire is shaped by the efficiency of β-selection and both the TCRα and TCRβ, by the efficiency of positive selection, alterations in the repertoire may reinforce the findings on β-selection and positive selection.

**Figure 5:**
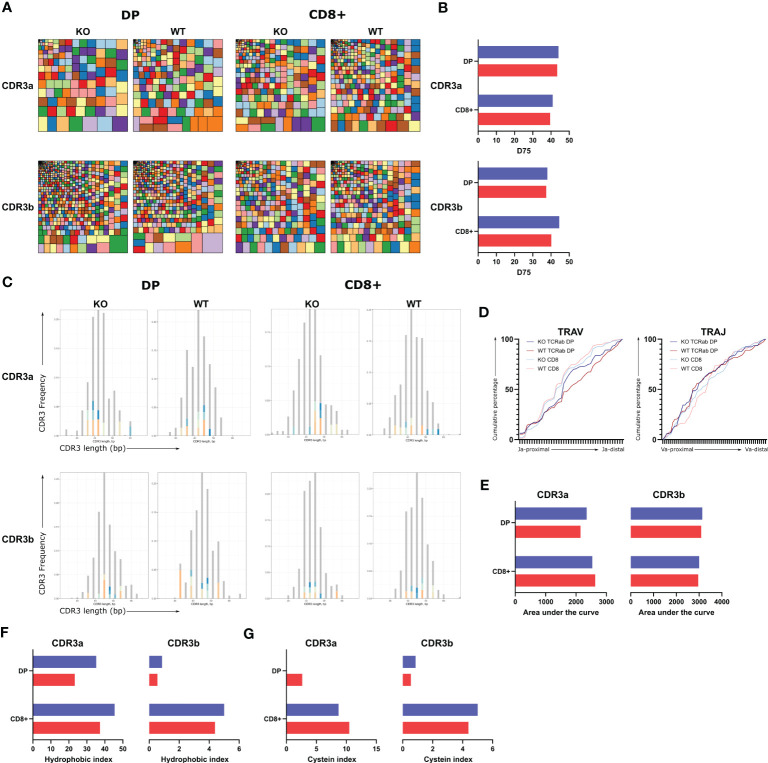
TCR repertoire of WASexon2-PGK-GFP (KO) or WASexon2-12-PGK-GFP (WT) derived GFP+TCRαβ+CD3+ DP and GFP+TCRαβ+CD3+ CD8+ cells in T cells generated in ATO cultures. **(A)** Tree maps displaying CDR3α (above) and CDR3β (below) repertoire clonality for KO (left) and WT (right) DP and CD8+ samples sorted from ATO cultures at 9 weeks. Each square represents one CDR3 clonotype and its size corresponds to its relative frequency in the repertoire. Colours were allocated randomly. **(B)** D75 analysis comparing the KO and WT samples. **(C)** CDR3 lengths of clonotypes are plotted according to their frequency (spectratyping plots) from TCRα (top) and TCRβ(bottom) repertoires of KO (left) and WT (right) DP and CD8+ cells. The top 10 most abundant clonotypes are colored. **(D)** Cumulative percentage of TRAV (left) or TRAJ (right) gene segment usage. X-axis represents the location in the TRAV or TRAJ locus. **(E)** Area under the curve determined from the cumulative plots from each sample for TRAV and TRAJ. **(F, G)**. Percentage of unique sequences with self-reactive hydrophobic doublets at position 6 and 7 of CDR3 (hydrophobic index, **Figure 4F**) or percentage of unique sequences with cysteine within 2 positions of the CDR3 apex (cysteine index, **Figure 4G**) for CDR3α (left) and CDR3β (right). Colour legend for **Figure 5** is as follows: KO = Blue; WT = Red.

ATO cultures were initiated with *WAS*_exon2_-PGK-GFP (KO)– or *WAS*_exon2-12_-PGK-GFP (WT)–transduced HSPCs. At week 9, TCRαβ^+^CD3^+^DP GFP^+^ and CD8^+^ SP GFP^+^ were sorted from ATO cultures, resulting in following populations: KO DP, KO CD8^+^, WT DP, and WT CD8^+^. Tree map representation and quantification by the D75 measurement of evenness/diversity indicated that deletion of WASp (KO) did not influence the diversity of the TRA and TRB repertoire present in the DP and the CD8^+^ SP populations (**Figures 5A, B**), which was in line with the Gaussian distributions of the CDR3 lengths of the TRA and TRB repertoires (**Figure 5C**).

TCRα rearrangements occur in an ordered fashion: J proximal V segments (*TRAV39*, *TRAV40*, and *TRAV41*) and V proximal J segments (*TRAJ61*, *TRAJ59*, and *TRAJ58*) are used first during the DP stage. As the DP cells mature, J distal V segments and V distal J segments are used. Because we observed a partial block at the DP to SP stage, we investigated whether V segment usage was affected in KO T lineage cells. However, no obvious differences were seen, neither at the DP stage nor at the CD8 SP stage (**Figures 5D, E**). Finally, as autoimmunity is part of the WAS phenotype, we analyzed the repertoire for autoreactive features (28). No differences were found either in the percentage of TCRs with hydrophobic doublets or in the TCRs with apical cysteines, which are characteristics for autoreactive T cells (**Figures 5F, G**). In conclusion, we did not find evidence for defects in the TCR repertoire being induced during T-cell ontogeny upon deletion of WASp.

## 4 Discussion

Patients with WAS often display T-cell lymphopenia ​ (31, 32)​. Although patients with WAS show a normal amount of circulating naïve CD4^+^ T cells, the number of CD8^+^ T cells is low compared with that in healthy individuals ​ (33)​. Although there is a limited number of reports published on the role of WASp on T-cell ontogeny in WAS^-/null^ mice ​ (8, 34, 35)​, induced pluripotent stem cells ​ (19)​, and patients with WAS ​ (9, 10, 31), this work represents the first comprehensive study on the influence of WASp on T-cell development in human.

Our strategy used ATOs as a substitute for intrathymic T-cell differentiation. Seet et al. have shown that the use of ATOs was a good and reliable tool for the study of human T-cell differentiation ​ (22)​. Indeed, Notarangelo and colleagues showed that the ATO system could help to determine the exact stage at which T-cell development is inhibited in certain T-cell–related diseases and establish whether the T-cell deficiency in patients reflects intrathymic abnormalities ​ (29). Using this strategy, we observed a partial block at the positive selection checkpoint, especially on the generation of CD8^+^ SP cells. This blockade was evidenced both by the inverted CD8^+^ SP to CD3^+^ DP ratio in KO T cells as well as by the increased outgrowth of WT T-cell precursors at the CD8^+^ SP stage as judged from the decrease of indels in this population. This is in accordance with the observation that less naïve CD8^+^ SP T cells are present in patients with WAS ​ (33)​. A more pronounced but qualitatively similar picture was observed with cell knocked out for IL2RG, the defective gene in X-SCID. X-SCID patients have a defect in the IL-7 receptor signaling pathway, which is involved in TCR rearrangements and T-lineage commitment. As expected from published data ​ (20, 29, 30)​, we saw a drop in the indel frequency going from the CD34^+^ to the CD7^+^CD5^+^ stage. The CD7^+^CD5^+^ cells just start to express CD1a and are considered the first T lineage–specific precursors (36). On the basis of these control data, we conclude that the decrease in indel frequency seen during the WASp KO T-cell differentiation is caused by the absence of WASp. Indeed, the decrease in indels going from the TCP1 stage to the CD4^+^ SP and CD8^+^ SP stage was statistically significant.

In addition, we could confirm the influence of WASp on positive selection in humanized NSG mice. Flow cytometry data and, again, the use of indel frequencies showed that WASp promoted the differentiation from CD3^+^ DPs to SP cells in the thymus, especially for the CD8^+^ SP cells. In the thymus, this defect was only partial and was further emphasized in the spleen. A possible explanation can be found in the reduced proliferative ability of WASp-deficient T cells ​ (32)​. Finally, GFP^+^ WAS–edited KO cells again showed an inverted CD8^+^ SP to DP ratio compared with GFP^+^ WAS–edited WT cells. All these data suggest that the lymphopenia observed in patients with WAS is, in part, the result of defective thymic output and, in part, resulting from defective homeostatic peripheral expansion. Interestingly, the eGFP^+^ KO cells expressed less CD69, a marker for positive selection. Furthermore, the generated KO CD8^+^ SP T cells were more immature, compared with the other populations where more than 90% of cells were CD27^+^CD1a^−^. Post-selection CD69^+^ thymocytes first acquire CD1a and then CD27 and, subsequently, lose CD1a during maturation ​ (36)​. CD27^+^CD1a^−^ cells require signaling through the TCR-MHC complex and CD27 to survive ​ (37, 38)​. At the site of priming, CD27 promotes the survival of T cells throughout consecutive rounds of proliferation ​ (38)​.

Laskowski et al. ​ (19)​ showed that WAS KO iPSC compared with WT iPSC cells generated few CD4^+^CD8^+^ DP and few mature CD3^+^ T cells in OP9-DL1 monolayer cultures (19). These data suggest an earlier blockage already at the β-selection stage. This discrepancy could be attributed to the fact that, in these studies, iPSCs were used rather than HSPCs. iPSCs are known to be very sensitive cells, and their culture conditions need to be optimal for the generation of multipotent HPSCs. In addition, iPSCs suffer from batch-to-batch variability and co-occurrence of heterogeneous populations of lineage subtypes and/or non-relevant cells as contaminating cell populations and variability in differentiation potential, tumorigenic potential, immunogenic potential, epigenetic status, and maturation characteristics ​ (39)​. All of this may have caused the WAS KO iPSC to differ from the WT iPSC, although being derived from the same patient, not only in the WAS gene but also in other aspects. Alternatively, these differences could also be explained by the fetal-like intrinsic nature of iPSC-derived hematopoietic cells that may lead to a T-cell differentiation differentially regulated compared with the one of postnatal T-cell differentiation ​ (39–41)​. Finally, the WAS iPSCs contained a dominant negative mutation in the WAS gene, whereas, in our study, this was not the case. Specifically, the mutation present in the iPSC study is an 1305insG WAS mutation. This yields a frameshift at amino acid 424 leading to the early termination at position 493, giving rise to a truncated version of WASp ​ (42)​. This truncated version is terminated before the VCA domain, important for ARP2/3 binding and actin filament formation. Studies in mice showed that the presence of a truncated WASp in WAS^−/−^ mice leads to a more severe phenotype ​ (43)​. Indeed, it was suggested that the truncated WASp competes for upstream effector proteins with other VCA-containing proteins, such as N-WASp that has redundant functions for WASp. A N-WASp/WASp double KO generates the same phenotype as the WASΔVCA mice ​ (8)​.

Literature describes a skewed TCR repertoire in patients with WAS ​ (13)​. Here, we observed a polyclonal TCRα and TCRβ repertoire in the KO DP and CD8^+^ SP populations generated in ATO cultures. These findings suggest that extrathymic clonal expansion rather than limited thymic output is the cause of the repertoire abnormalities. Studies in WAS^−/−^ mice showed a TCRβ repertoire that was indistinguishable from littermates’ controls ​ (44)​. This was confirmed in patients with WAS, showing that mainly older patients have a skewed repertoire. Wada et al. showed that only younger patients had a normal TCRβ repertoire, suggesting that thymic diversification was intact ​ (45)​. It was reasoned by the authors that the oligoclonal repertoire may be the result of chronic infections or accumulation of T-cell clones specific to autoantigens. On the contrary, O’connell et al. showed that even young patients had an oligoclonal repertoire ​ (13)​. However, this could be caused by a decreased survival of T cells in the peripheral organs. Furthermore, O’connell et al. showed that clonal expansions were more prominent in patients with somatic reversion and/or chronic infections, intensifying the hypothesis that the skewed TCR repertoire seen in patients with WAS is generated extrathymically ​ (13)​.

In summary, by using CRISPR-generated WAS KO cells and extensive testing in *in vitro* ATO cultures as well as *in vivo* in humanized NSG mice, we have shown that WASp is required for positive selection (and differentiation) to SP T cells. The defect is partial and does not interfere with the generation of a rich TCR repertoire during T-cell development, suggesting that the skewed TCR repertoire in patients with WAS is due to extrathymic clonal expansions. Along with recently published data ​ (13, 46, 47)​, this study may provide a framework and novel starting point for WAS disease correction.

## Data availability statement

The datasets presented in this study can be found in online repositories. The names of the repository/repositories and accession number(s) can be found below: PRJNA962053 (SRA).

## Ethics statement

The animal study was reviewed and approved by Ethics Committee of the Faculty of Medicine and Health Sciences, Ghent University, Ghent.

## Author contributions

Conceptualization: MP and BV; methodology: MP and BV; investigation: MP, GS, and GG; formal analysis: MP and GS; resources: TT, GL, BD, and BV; supervision: TK and BV; writing—original draft preparation: MP and BV; writing—review and editing: MP, JA, GS, SM, HJ, LB, KW, SB, HX, JI, LC, EP, LD, DV, GL, TK, BD, and BV; visualization: MP; funding acquisition: MP and BV. All authors have read and agreed to the submitted version of the manuscript.
