# Supplementary material for: The Wiskott–Aldrich syndrome protein is required for positive selection during T-cell lineage differentiation
Source: Front Immunol. 2023 Jun 7;14:1188099. doi: 10.3389/fimmu.2023.1188099 (PMC10282776; doi:10.3389/fimmu.2023.1188099)
Supplement: Supplementary file 1 [file DataSheet_1.docx]

# Supplementary Figures and Tables

## Supplementary Table

**Table S1. Forward, reverse and sequencing primer for each gRNA**

|  | gRNA (5’-3’) | Forward primer (5’ – 3’) | Reverse Primer (5’ – 3’) | Sanger Sequencing Primer (5’ – 3’) |
| --- | --- | --- | --- | --- |
| I1 | CATGACAGTCATGGGCCCAA | ACCATGAGTGGGGGCCCAAT | TGGTTCTGGGGCTCAGGGAT | ACCATGAGTGGGGGCCCAAT |
| E2 | CTGGACCAAGGAGCATTGTG | ACCGTTTCTTCCTCTTCCTCTCC | GCACTGTATTTGTACCTGAACCT | ACTTGCCTTCCCTCTGG |
| IL2RG | GGTAATGATGGCTTCAACA | ACCACCTTACAGCAGCACC | ATGATGGTCAGAAGGAGGAGG | AGGACTTAGCCCGTGTC |

## Supplementary Figures


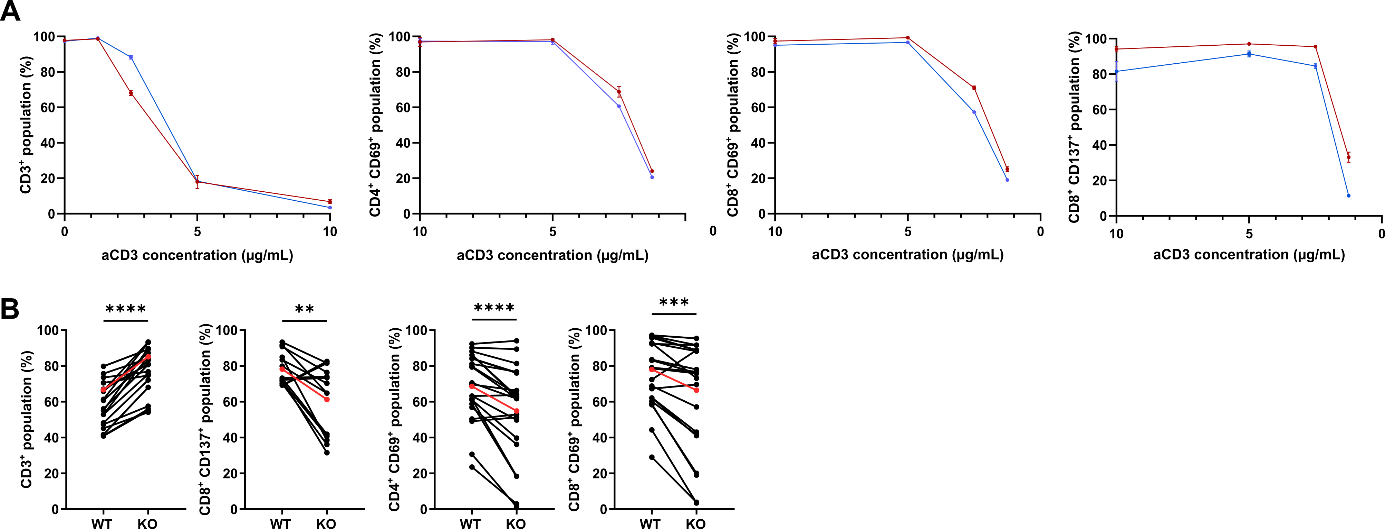


**Figure S1. CD3 downmodulation and CD69 and CD137 upregulation in I1 and E2 T cells
A.** Representative curve showing CD3 downmodulation and CD69 and CD137 upregulation after 6h stimulation with various concentrations of aCD3 and 10µg/mL aCD28. Show is the mean amount of CD3/CD69/CD137 positive I1 (WT = Red) and E2 (KO = Blue) total, CD4+ or CD8+ T cells. **B.** CD3 downmodulation and CD69 and CD137 upregulation in I1(WT) and E2 (KO) T cells after 6h stimulation with 2.5µg/mL aCD3 and 10µg/mL aCD28. (n=11 experiments from 7 donors with 2 technical replicates; **p<0.01, ***p<0.001, ****p<0.0001; two-tailed paired t test). Red points represent the mean.


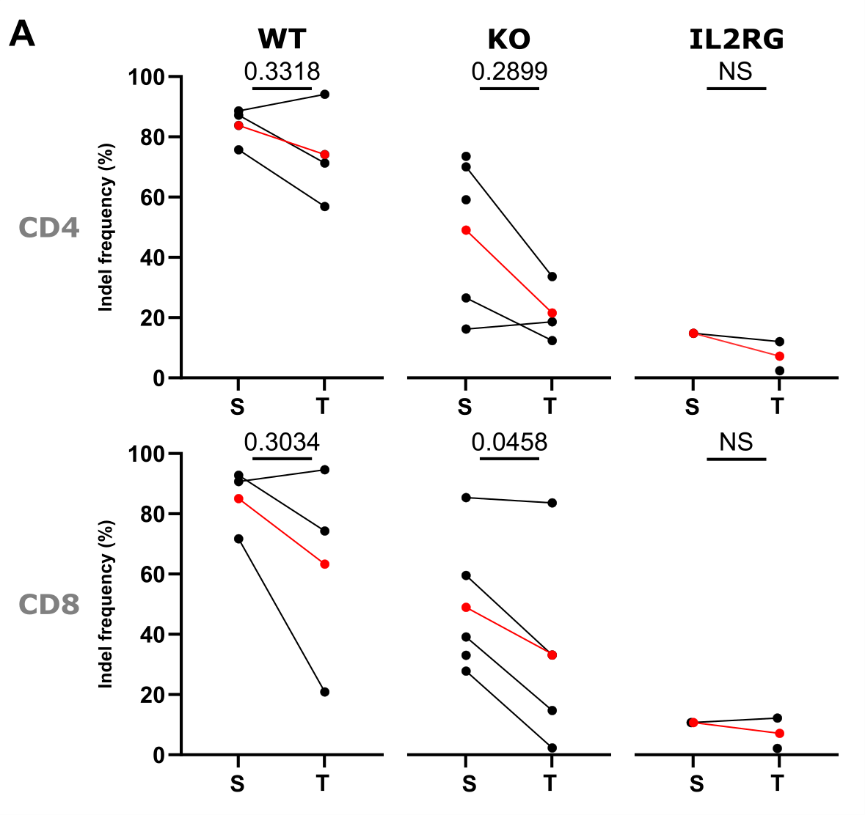


**Figure S2. Comparison of indel frequency in CD4^+^ SP and CD8^+^ SP cells in spleen and thymus
A.** Indel frequencies in the spleen (S) (left) and thymus (T) (right) stage for WT, KO and IL2RG in the CD4^+^ SP and CD8^+^ SP cells (n=3 for WT, n=5 for KO and n=2 for IL2RG; exact p values in Figure; NS = not able to be determined; two-tailed paired *t* test). Data in Figure S2 are presented by mean ± SEM. Red points in G and H represent the mean. Single points have no matched value.
